# Effect of Ranque-Hilsch Vortex Tube Cooling to Enhance the Surface-Topography and Tool-Wear in Sustainable Turning of Al-5.6Zn-2.5Mg-1.6Cu-0.23Cr-T6 Aerospace Alloy

**DOI:** 10.3390/ma15165681

**Published:** 2022-08-18

**Authors:** Jasjeevan Singh, Simranpreet Singh Gill, Manu Dogra, Shubham Sharma, Mandeep Singh, Shashi Prakash Dwivedi, Changhe Li, Sunpreet Singh, Shoaib Muhammad, Bashir Salah, Mohamed A. Shamseldin

**Affiliations:** 1 Research Scholar, IKGPTU, Kapurthala 144603, Punjab, India; 2 Department of Mechanical Engineering, Sardar Beant Singh State University, Gurdaspur 143521, Punjab, India; 3 Department of Mechanical Engineering, Punjab University SSG Regional Centre, Hoshiarpur 146021, Punjab, India; 4 Department of Mechanical Engineering, IK Gujral Punjab Technical University, Main Campus-Kapurthala, Jalandhar 144603, Punjab, India; 5 Department of Mechanical Engineering, University Centre for Research and Development and Chandigarh University, Mohali 140413, Punjab, India; 6 School of Mechanical and Mechatronic Engineering, Faculty of Engineering and Information Technology, University of Technology Sydney, 15 Broadway, Ultimo, NSW 2007, Australia; 7 G.L. Bajaj Institute of Technology and Management, Greater Noida, Gautam Buddha Nagar, Greater Noida 201310, Uttar Pradesh, India; 8 School of Mechanical and Automotive Engineering, Qingdao University of Technology, Qingdao 266520, China; 9 Department of Mechanical Engineering, National University of Singapore, Singapore 119077, Singapore; 10 Institute of Manufacturing, Engineering Management Program, University of Engineering and Applied Sciences, Swat 19060, Pakistan; 11 Industrial Engineering Department, College of Engineering, King Saud University, P.O. Box 800, Riyadh 11421, Saudi Arabia; 12 Mechanical Engineering, Faculty of Engineering and Technology, Future University in Egypt, New Cairo 11835, Egypt

**Keywords:** Al 7075-T6, turning, surface roughness, SEM analysis, microhardness, RHVT, tool wear, chip management model, energy-consumption

## Abstract

The aerospace metal cutting industry’s search for environmentally friendly practices that do not compromise machining performance is well known. One of the major objectives is the reduction in use of cutting fluids, which play a major role in containing the harsh effects of severe heat generated during machining. Machining performance and product quality can be improved by controlling heat during machining. The purpose of this study was to determine the effectiveness of various environmentally friendly metalworking fluid (MF) strategies for the sustainable turning of aerospace aluminum alloy (Al-5.6Zn-2.5Mg-1.6Cu-0.23Cr-T6) for automotive, marine, and aerospace industrial applications. The SEM images were analyzed for worn tool surfaces and machined surfaces. Under dry conditions, heat does not dissipate well, and will enter the workpiece due to the absence of coolant. This causes extreme damage beneath a turned workpiece. Thus, at 10 µm, a drop in microhardness of approximately 20% can be observed. A similar observation was made in a Ranque-Hilsch vortex tube (RHVT) and in compressed air; however, the drop in hardness was relatively low compared to dry conditions. This evaluation of microhardness indicated a heat-based attention in the turned workpiece, and thus, the heat-based effect was found to be lowest in RHVT and compressed air compared to dry conditions. Results showed that RHVT reduces temperature up to 10%, surface roughness 13%, and tool wear 20% compared to dry turning. Overall, RHVT was identified as more effective environmentally friendly cooling strategy than dry and compressed air for the turning of aluminum alloy 7075-T6.

## 1. Introduction

Nowadays, an assortment of cutting coolants are employed by the metal cutting industry, the use of which help to dwindle friction, wear, and cutting temperature, and can assist with chip removal in the cutting area. The use of cutting coolants improves surface roughness and extends the working lives of tools. Due to environmental concerns, environmentally responsive cooling and lubrication conditions such as compressed air, dry environment, and RHVT are becoming popular. The advantages of these conditions include: a lesser amount of pollution to the environment, cleaner chips, a reduction in disposal costs, and an improvement in harmlessness to skin [1]. Air-cooling has not yet been extensively explored for machining industry use compared to the use of traditional cutting fluids [2]. The use of a RHVT to provide cold air to cool the tool interface in machining showed encouraging results for improving the performance of machining through air-cooling [3]. In order to establish RHVT technology as an effective cooling technology for the machining of materials, the optimization of RHVT geometrical parameters and fluid parameters is needed. This issue has not yet been deeply explored by turning operation researchers.

An RHVT-assisted MQL method has been used in assorted machining processes, namely grinding, milling, turning, and drilling [4,5]. An intensive review of the benefits of employing cold compressed air in a variety of machining operations has been performed. It was reported that use of compressed air showed potential to reduce tool wear, friction, and cutting force [6]. Rodzi et al. [7] carried out the turning of cast iron FCD 700 and compared the machining performances under dry environment and chilled air. They concluded that chilled air improved the tool life. The use of RHVT enhanced the effectiveness of cooling on the cutting zone and also led to reduced carbon emission compared to dry turning. Further cooling performance under RHVT was comparable to that of MQL and the generation of PM2.5 was almost negligible under RHVT compared to MQL [8]. Dry machining is an environmentally safe and sustainable cooling and lubrication method, and can be effectively used in machining with the selection of suitable tools and cutting conditions [9]. However, there are many significant issues associated with dry cutting that adversely affect machining performance. The effectiveness of cutting zone cooling under dry turning is poor, which restricts the use of dry turning for various materials under different combinations of tuning parameters. Thus, the restricted use of dry turning opens the door for researchers to explore the exclusive use of air (under compressed state, along with RHVT) as a sustainable cooling technology for the turning of various materials [10]. However, there are many significant issues associated with dry cutting that adversely affect machining performance. Aluminium is an important metal that plays a significant role in the world economy, and is widely employed by industries that manufacture different kinds of products [11,12]. Owing to its lightweight and mechanical properties, aluminium alloys find broad applications in the aerospace, defense, and automotive industries [13,14].

The manufacturing sector is constantly looking for new technologies that can help manufacture quality products by minimizing costs, reducing the consumption of materials, and reducing the amount of cutting tools needed. Hence, it is essential to determine suitable cutting parameters (machining environments, cutting speed, feed, force, depth of cut, and number of passes). Preceding studies have shown that tool life and workpiece quality are affected by many situations and parameters [15]. Cutting fluids are chosen to avoid problems on part surfaces and to reduce high temperatures in order to protect tools during manufacturing operations. Timely maintenance of the coolant is required. Coolant can be degraded by the formation of bacteria, by reaction with oil, and by evaporation of the water it contains [16,17]. These deteriorations have a negative impact on human health, the environment, the workpiece, and the tools themselves, so the use of green coolant/lubrication strategies is vital. In conventional flood cooling, the effectiveness of cooling is comparatively high compared to the use of compressed air and RHVT as cooling technologies in machining. However, the environmental concerns have increased the cost of waste fluid treatment in conventional flood coolant systems. With the exclusive use of air as a coolant through RHVT/compressed air, the effectiveness of cooling is not the same as that of flood cooling, but the environmental impact and overall process cost is very low compared to conventional flood cooling [18]. If emulsion- or soluble-type metalworking fluids continue to be used, more expensive treatment facilities will be needed in order to reach a zero-emission standard. Thus, these ecological issues, as well as the issues of cost and human health, encourage the use of air as a coolant in turning as a sustainable cooling strategy.

Machining of Al7075T6:

The goal of sustainable strategies in Al 7075-T6 machining is fairly important for achieving viability in terms of the economic and environmental aspects. The Al 7075-T6 alloy is a hard aluminum alloy that belongs to the Al–Zn–Mg–Cu alloy family. When heated, it exhibits excellent strengthening properties. As a result, it is widely used in the aerospace industry as well as for high strength structural components. The value of Al 7075-T6 in the aerospace and automotive industries is well known. This alloy frequently requires machining to achieve the desired shape and size. The main issue with machining Al 7075-T6 is material adhesion on the tool–work interface, which results in built-up edge [19]. Material removal rate increases as jet pressure, abrasive particle size, and exposure time increase, but decreases as standoff distance increases while machining Al 7075T6 alloy, reported by Nyaboro et al. (2021) [20]. The inclusion of the environmentally friendly strategies MQL and cryogenic can improve surface finish and improve chip evacuation [21].Vasu et al. (2021) conducted cutting of Al 7075T6 alloy and observed the chip’s saw-tooth appearance grows as the feed rate increases. The serrations on the chip also increase as the cutting speed increases. The experimental and numerical analysis of chip morphology revealed that chip thickness increases with increasing speed and feed rate. [22]. A three-axis milling tool dynamometer is used to measure cutting forces. ANOVA is used to assess the model’s adequacy. According to the findings, feed rate has the greatest statistical and physical influence on cutting force. [23].

However, there are some disadvantages to machining aluminium alloy, such as adhesion wear and built-up edge (BUE) formation, which can reduce tool life. Machining performance, surface roughness, and cutting tool life all suffer as the tool wears down. Many studies have been conducted in order to minimize this critical issue. The purpose of this project was to investigate the cutting tool performance of a coated carbide tool in turning operation on Al 7075-T651, including the tool wear rate, energy consumption, microhardness, surface roughness, and chip morphology.

### 1.1. Sustainable Manufacturing 

Green manufacturing is the manufacturing of products that use environmentally friendly techniques during manufacturing to lessen the effect on environment, save energy, and increase worker and community safety. To protect the environment, all manufacturing organizations should aim for activities that lessen the use of water and reduce energy consumption, waste generation, and emissions. Waste generation should be minimized and is not preferred in metal machining as it affects the health of workers, reduces productivity and negatively impacts the environment. The development of new, green processes or the optimization of existing processes to generate fewer emissions and employ less harmful materials should be considered. Because of our shared responsibility for the world in which we live, the adoption of sustainable manufacturing practices is of crucial importance for all production engineers. To overcome environmental problems, eco-friendly technologies that minimize metal working fluid (MF) consumption during machining are being developed.

**Figure 1:**
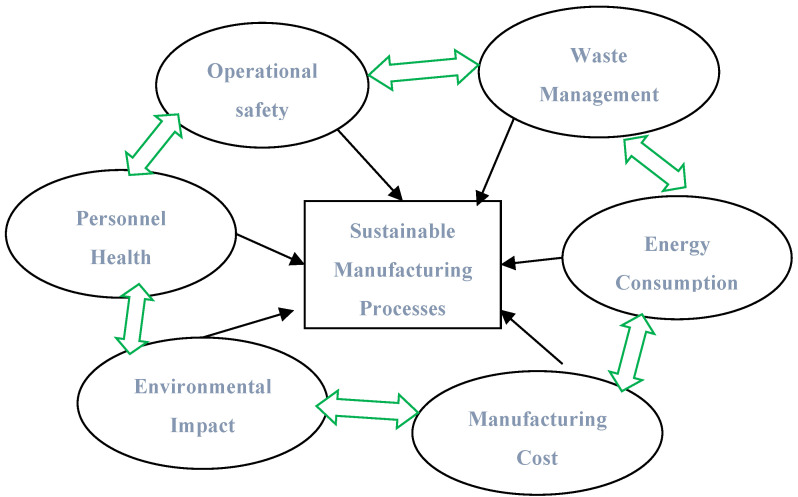
Six Elements of Sustainable Manufacturing Processes.

Today, manufacturing industries are adopting a reduction in metal working fluid to achieve sustainability goals with respect to social, economic, and environmental concerns. Six key elements affect the sustainability of manufacturing processes, as depicted in Figure 1. Environmentally friendly cooling lubricating/conditions such as MQL and dry machining are widely used by manufacturing industries due to environmental regulations. Sustainable manufacturing can be achieved through the use of processes that have fewer environmental concerns, conserve energy, and are safe for consumers and communities. In dry machining, no cutting oil is used, so the sustainability of dry machining is high, but a modification of dry machining is recommended due to limitations such as high tool wear, built up-edge, high friction, and increased surface roughness.

### 1.2. Cooling by RHVT

**Figure 2:**
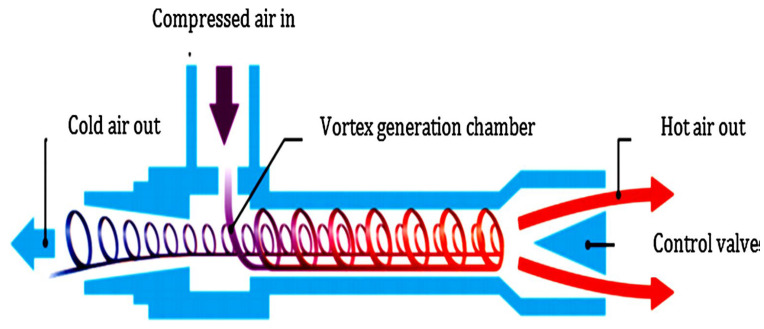
Generating of cold air employing RHVT [24].

RHVT has been employed in a diversity of applications such as metal cutting operations, automotive engine manufacturing, machine element cooling, and in nuclear reactors. Figure 2 depicts the schematic image of RHVT, and fluid has combinations of both axial and rotational motion. Compressed air at higher pressure enters the RHVT tangentially, resulting in the formation of a vortex flow in the tube, which emerges from the openings at the two ends. The air is released at considerably lower as well as higher temperatures from both the cold and hot ends of the RHVT, respectively [24].

### 1.3. Shortcomings of Previous Research Studies

Use of a Ranque-Hilsch vortex tube (RHVT) as a reliable coolant technology has not been deeply explored. Research on the use of RHVT along with optimized cutting parameters like speed, feed & depth of cut, coupled with various cutting tool/workpiece material combinations is scant RHVT parameters such as dimensions and inlet pressure for compressed gas or air must be thoroughly investigated before their full potential can be realized. The use of RHVT under MQL has also not been deeply explored, and there is still much to know about the potential for RHVT technology to be used as a sustainable cooling method in machining. Furthermore, there is a scarcity of RHVT research on workpiece material hardness under various workpiece material/tool material combinations. Further studies pertaining to a comprehensive evaluation of the performance of RHVT in terms of tool temperature, cutting forces, tool wear, surface roughness, and micro-hardness variation as an indicator of surface integrity have not yet been performed.

### 1.4. Motivation for Present Work

In light of Section 1.3, an exploration of the potential for RHVT to be used as a suitable cooling technology in the turning of Al 7075-T6 was undertaken. The significance of Al 7075-T6 in the aerospace and automotive industries is well understood. There is a common desire to machine this alloy to obtain a preferred form and size. The key problem related to the machining of Al 7075-T6 is the adherence of material to the tool–work interface, resulting in built-up edge. However, as mentioned earlier, we want to eliminate the use of coolant in order to better understand sustainable machining practices for Al 7075-T6. A comparative assessment of the impact of MF techniques on environmental sustainability vis-à-vis overall machining performance has not yet been made. Thus, in the current work, in depth assessment of dry machining, Ranque-Hilsch vortex tube (RHVT), and the use of compressed air as MF for the turning of Al 7075-T6 with carbide tools have been performed to investigate machining overall performance and power consumed. From the previous research shortcomings, it is clear that the amount of studies evaluating the turning performance in terms of tool wear, tool temperature, cutting forces, surface roughness, and micro-hardness variations using RHVT is limited. Therefore, in this study, the overall turning performance of all three MF techniques was evaluated in terms of tool tip temperature, surface roughness, chip morphology, tool wear and microhardness. This study will create more insight related to sustainable cooling technology, the use of air as a cooling medium in the machining of materials with proper tool selection, and the combination of cutting parameters with respect to the material used.

## 2. Experiment Setup

**Figure 3:**
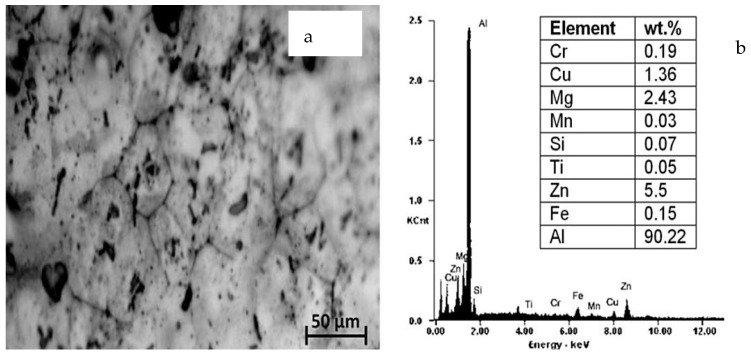
(**a**) SEM image showing microstructure; (**b**) EDS showing chemical composition of Aluminium alloy 7075-T6.

**Figure 4:**
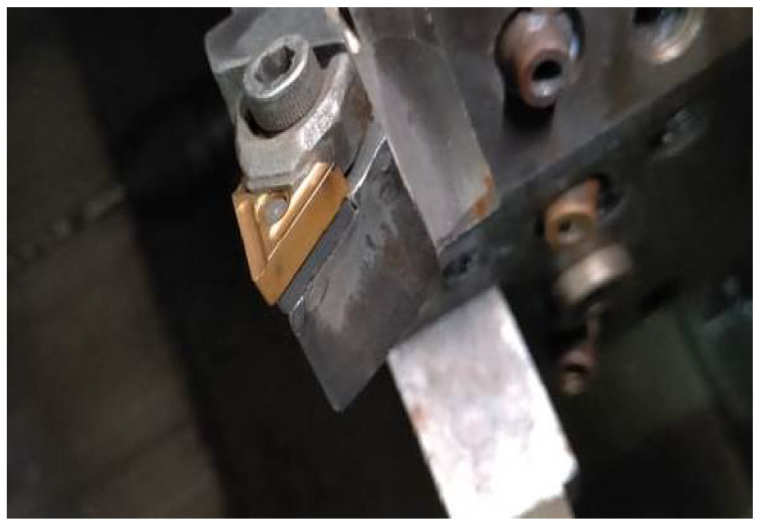
Cutting insert used.

**Figure 5:**
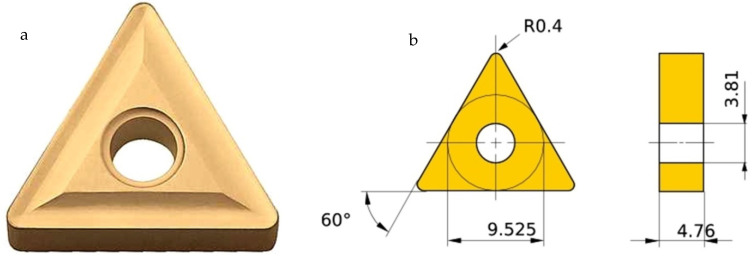
(**a**) Pictorial view; (**b**) dimensions (mm) of the insert (TNMG160404L-ST) used for turning.

**Figure 6:**
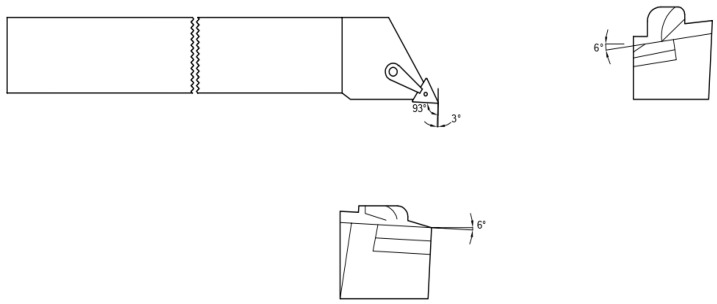
Cutting insert showing rake angle.

**Figure 7:**
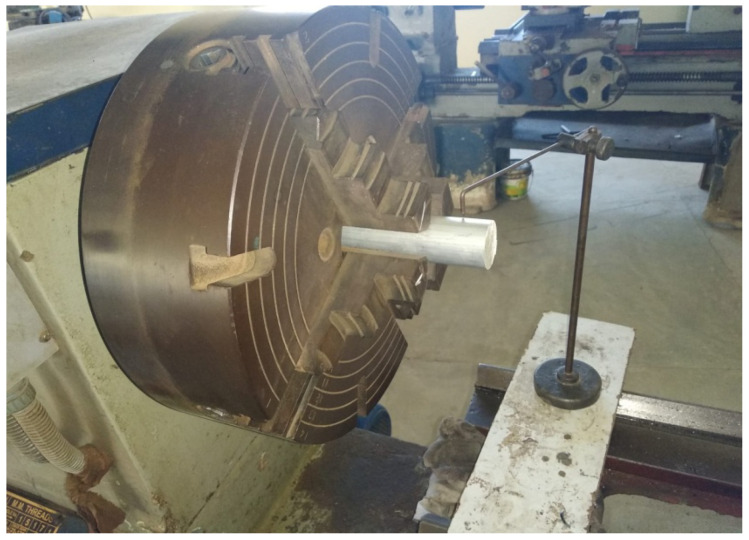
Centering of workpiece.

**Figure 8:**
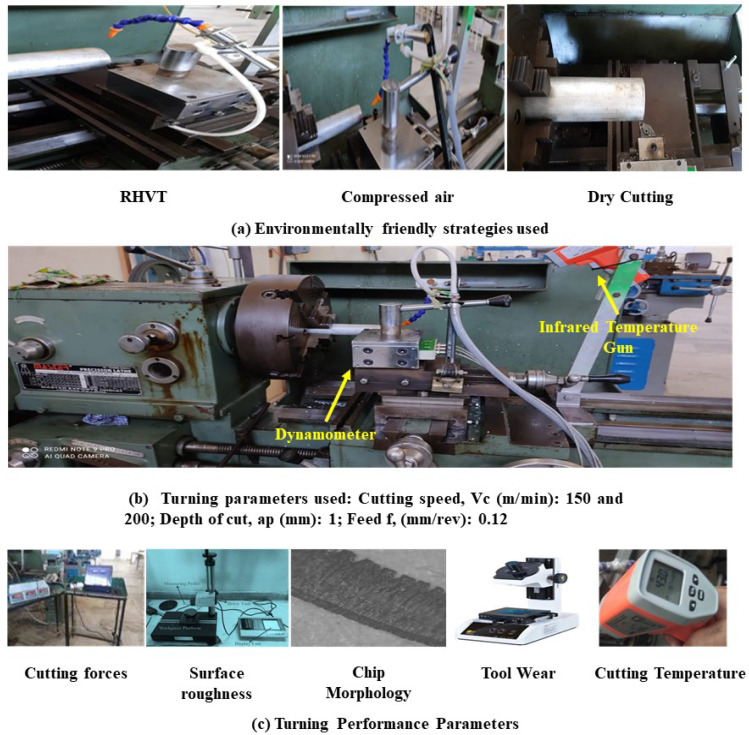
Experimental set-up. (**a**) Environment friendly conditions; (**b**) Turning parameters used: Cutting speed Vc (m/min): 150 and 200; Depth of cut, ap (mm): 1; Feed f, (mm/rev): 0.12 (**c**) Turning performance parameters.

A workpiece material Al 7075-T6 with a diameter of 38 mm and a length of 225 mm was used for the cutting experiments. Table 1 depicts the mechanical properties of the alloy. Energy dispersive X-ray spectroscopy (EDS) and scanning electron microscopy (SEM) were done to obtain the microstructure (Figure 3a) and chemical composition (Figure 3b) of aluminum alloy 7075-T6. The machine used for turning was a precision lathe machine (model Okuma LB15; spindle speeds 30–4200 rpm; power 15 hp), with depth of cut 1 mm, cutting speed m/min (150 and 200), with feed rate of 0.12 mm/rev. From the literature, it is quite evident that cutting speed is the most significant parameters affecting the turning performance, so cutting speed was varied in two steps on the higher side. Feed and depth of cut was kept constant on the basis of the literature, recommendation of tool manufacturers, and keep in the material removal aspects in mind. Three different cooling settings were tested, i.e., vortex tube air, dry (no air) and compressed air. To remove the consequences of tool wear, fresh cutting edge of insert was used for each test. The triangular shape cutting inserts TNMG160404L-ST was used and image and specifications are shown in Figure 4, Figure 5 and Figure 6 and Table 2. The insert was selected on the basis of literature and tool manufacturers’ recommendation under particular set of cooling techniques to be used. Centering of workpiece was accomplished with dial indicator to avoid inaccuracy during cut, as shown in Figure 7.The picture of experimental set-up is exhibited in Figure 8. Infrared temperature gun was used to measure tool tip temperature. It permits temperature measurements in the range of −55 °C to 910 °C, having resolution of 0.1 °C and an accuracy of ±0.74 °C. The cutting force (Fx, Fy and Fz) readings were directly recorded from dynamometer (Kistler make), having an accuracy of 0.05% on a full scale, calibrated and sampling at a frequency of ∼50 kHz. The surface roughness values were measured using surface profilometer (Mitutoyo-SJ-400) with high accuracy. 

As depicted in Figure 8, for the temperature measurement, a non-contact infrared optical probe was used. It measures temperatures from −50 °C to 900 °C with a resolution of 0.1 °C and an accuracy of ±0.75 °C. It was calibrated with infrared comparator cup before measuring the temperature. The distance-to-target ratio was considered less than 6 inches for measuring correct temperature. The sampling time chosen to measure tool temperature was proportional to the length machined. The mean values of the maximum temperature at the nose of the tool acquired in thrice-repeated experiments were taken as final cutting temperatures. 

Further, a commercially obtained three-component, digital lathe tool dynamometer, as shown in Figure 8, was also used to measure cutting forces. The charge amplifier was linked to a data acquisition system on the computer, which included an A/D converter and signal analyzer software. It measures cutting tool forces up to 500 kgf with a resolution of 0.1 kg. The mean values of cutting forces were recorded at regular intervals of time for analysis.

For analysis of surface roughness produced on the turned components, a Mitutoyo Surftest SJ-400 portable surface roughness analyzer (Figure 8). was employed to measure the roughness profile after each pass on the workpiece surface. Final surface roughness (Ra) values were obtained by averaging Ra results at a minimum of three locations on the workpiece surface.

For further analysis of surface morphology of turned components and chip analysis, as well as tool wear analysis, an (SEM-Model: JSM IT500) was used, as indicated in Figure 8. Scanning electron microscope helped in generating the high-resolution images at a magnification range from (5× to 500 and 1000×) of the machined surface, chips surface, and tool-worn portions.

**Figure 9:**
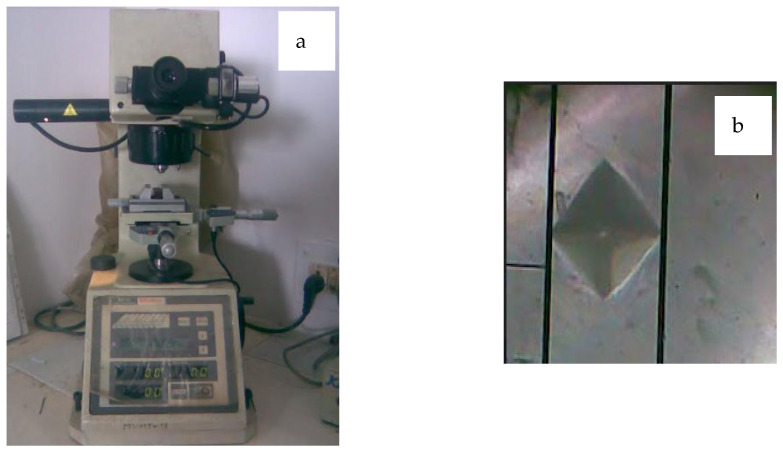
(**a**) Digital Micro-hardness Tester (**b**) Image of indent for micro-hardness analysis.

For Micro-hardness analysis on the turned surfaces, a Mitutoyo/HM 211 digital micro-hardness tester was used, as indicated in Figure 9a,b. The load control was automatic (Load, Dwell, Unload) with a Resolution of 0.01 µm. This measurement will help in arriving at a demarcation between machining affected zone and bulk material.

## 3. Results and Discussions

### 3.1. Tool Tip Temperature

**Figure 10:**
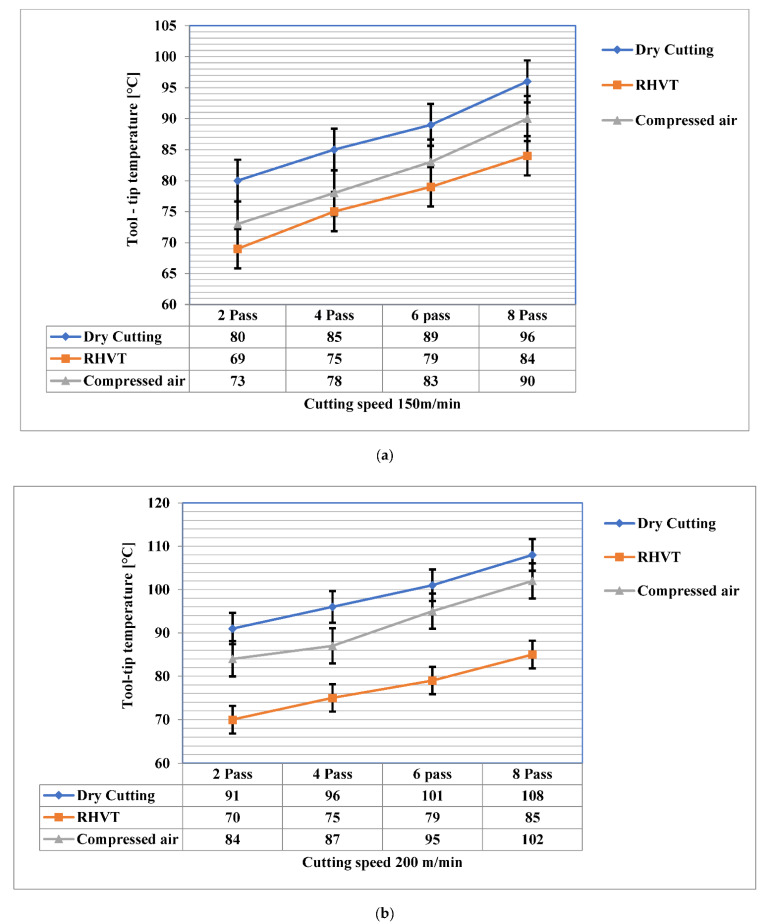
Effect on tool-tip temperature at cutting speed (**a**) 150 and (**b**) 200 m/min.

When cutting metal, the interface temperature between the cutting tool and the workpiece increases. Temperature affects not merely the wear rate of the cutting tool, but the surface integrity of the workpiece such as residual stress, hardness, and surface roughness as well. An infrared temperature gun was employed to measure tool tip temperature. It measures temperature from 55 °C to 910 °C with a resolution of 0.1 °C and an accuracy of ±0.74 °C. In this study, the tool tip temperature was measured with an infrared thermometer, and the result was shown in Figure 10 by measuring the average three times. Three different cooling settings were tested, i.e., vortex tube air, dry (no air), and compressed air. It is clearly visible from Figure 10 that the employment of cold air on the rake face of the cutting device decreases the temperature more effectively than dry conditions, which confirms the earlier findings [25,26,27]. The RHVT MF strategy demonstrates a significant drop in temperature compared to dry and compressed air conditions, as the high-velocity, low-temperature air supplied through the vortex tube cools the cutting area and effectively reduces the heat generated at the cutting edge. In addition, the temperatures recorded using compressed air was significantly lower than dry conditions, equivalent to the results acquired by Vanan et al. [28]. The temperature generated was higher under a dry environment due to the absence of coolant/lubricant. Irrespective of the cooling/lubrication method employed, Figure 10 shows that the temperature of tool tip increases owing to an increase in the strain rate as cutting speed increases. A similar result was obtained by Resis et al. [29].

### 3.2. Tool Wear

Machining productivity can be increased by extending the life of a tool. Tool wear affects tool performance. For both cutting speeds, tool wear rate was measured by using recorded data. In this study, tool flank wear was measured every minute with a digital microscope, and crater wear was analyzed with SEM. Based on tool life criterion, VB_max_ less than 600 microns was selected. The maximum flank wear (VB_max_) was recorded.

**Figure 11:**
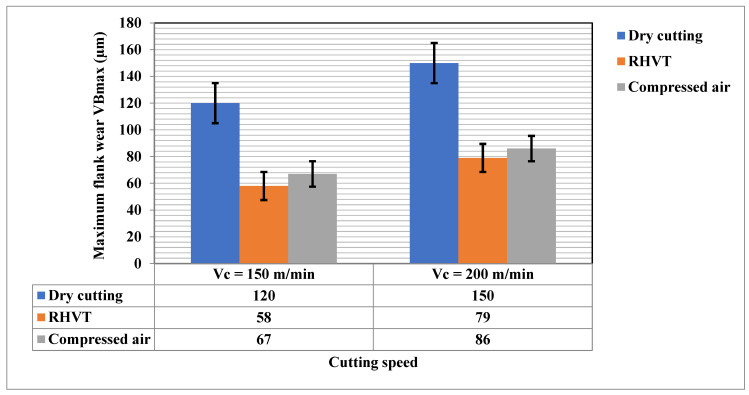
Depicts the consequence of cooling/lubrication environments on maximum flank wear VBmax at cutting speed Vc = 150 m/min, Vc = 200 m/min.

As per ISO 3665 standard, lives of cutting inserts can be assumed to depend on the rejection limits of wear parameters (any): average flank wear (≥0.3 mm), maximum flank wear (≥0.4 mm), notching (≥0.6 mm), nose wear (≥0.3 mm), and enormous chipping [26]. During the turning process, the flank face of a spindle is in direct contact with the worlpiece, causing steady wear as the contact surface undergoes considerable rubbing and friction [26]. The use of RHVT reduces flank wear better than dry conditions due to the chilling effect of air reducing the temperature [30,31,32]. Figure 11 shows that, by using RHVT and compressed air MF strategies, tool wear is reduced in comparison to dry cutting, leading to longer cutting life. Compressed air reduces tool flank wear by 75% and 80%, respectively, at cutting speeds of 150 m/min and 200 m/min in comparison to dry cutting, owing to its low temperature reduction in the cutting area. The MF dry-cut strategy was least effective, as it provided neither effective lubrication nor cooling.

The following behaviors are observed:

1. The VB_max_ decreases with the employment of RHVT better than dry and compressed air conditions.
2. The tool flank wear increases incrementally with cutting speed for the both cutting conditions.
3. RHVT is effective with each incremental decrease in cutting speed.

**Figure 12:**
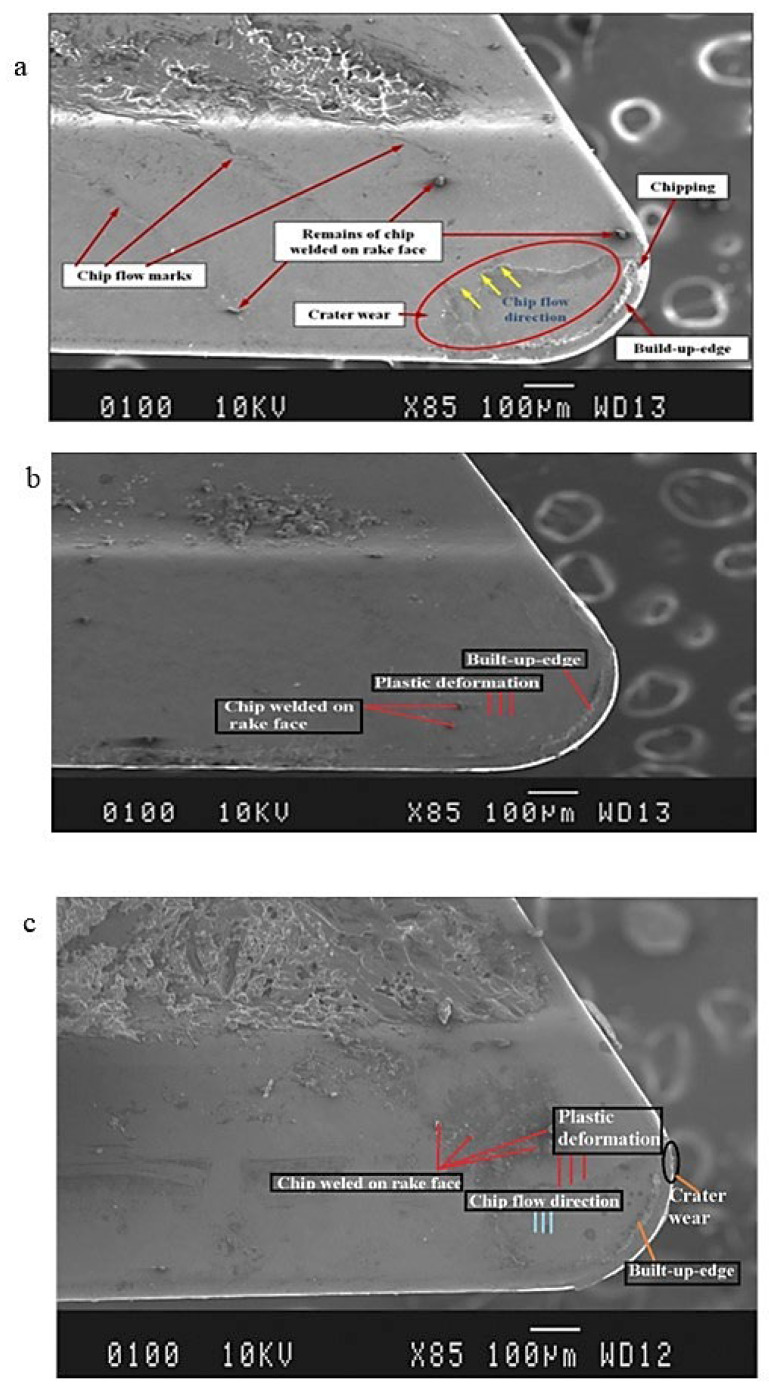
SEM pictures showing tool wear pattern for cutting speed of 150 m/min, (**a**) Dry; (**b**) RHVT; (**c**) Compressed air.

The SEM was used to investigate rake surface of tool subjected to dry, compressed air and RHVT conditions at speed of 200 m/min. In the case of Al 7075-T6 alloy, the essential task is to avoid chip welding on the rake face of the cutting tool because the presence of zinc makes this alloy prone to chip adhesion on the rake face. This can cause the formation of built-up edge (BUE), which raises the tool-chip interface temperature and ultimately reduces the length of tool life [33]. Hence, to minimize the wear and tear mechanism at the rake face of tool, the SEM perspectives of the worn tool have been acquired from the rake face, as exhibited in Figure 12. The flank wear of the cutting tools have been recorded quantitatively best and pictures of flank wear have been not acquired for the prevailing work.

Figure 12a shows the worn out tool insert after dry cutting. This depicts that the rake face has suffered severe crater wear. This can be seen at the relatively low 85× magnification. There is also some chipping close to the cutting edge of the tool. The occurrence of crater wear is associated with a low thermal coefficient of forced thermal effect during machining. This phenomenon occurred due to the presence of a sufficient amount of zinc in the Al 7075T6 alloy, causing the chip to easily weld to the rake face under high cutting forces. As a result, the increased cutting forces resulted in increased contact stresses during dry turning, resulting in crater wear. Jerold et al. [34] also reported that, in dry cutting, unacceptable cutting temperatures without cooling and lubricating effects lead to crater wear and BUE during machining. The reported chipping near the cutting edge was found to be due to the tool softening at high temperatures, as carbide tools have a low softening point of 1100 °C [35].

An SEM image taken using RHVT MF strategy shows minor BUE, shown in Figure 12b.This shows that there was little chip adhesion on the tool surface owing to lack of lubrication in the RHVT MF strategy, which shows less tool wear compared to the dry cutting MF strategy. Plastic deformation on the rake surface is less compared to dry cutting, as can be seen clearly in the image. Although the supply of cooled air could limit the increase of temperature at the chip tool interface, it was still not sufficient to completely prevent chip welding on the rake face.

Figure 12c discloses the existence of significant crater wear, chip welding on rake face, BUE, and plastic deformation, nevertheless the overall wear of the rake face of the tool is less compared to the dry cutting environment. It may be because compressed air cannot provide a lubricating or cooling effect to the extent necessary to limit the formation of crater wear, BUE, plastic deformation, and chip welds on the rake face.

From the above information on tool wear, it can be deduced that the flank and rake face wears using RHVT fluid strategy were significantly less compared to the compressed air and dry cutting strategies. Since plastic deformation is directly related to temperature increase, the presence of severe plastic deformation for the dry cutting and compressed air MF strategies clearly indicates elevated temperature at the tool chip interface. This observation is consistent with an upward trend in tool tip temperature as discussed in Section 3.1. The productivity of the cutting tool using the RHVT MF strategy was quite satisfactory, allowing it to be used for oil-free Al 7075T6 turning. This is how the machining environment can be made safe and sustainable at the expense of tool performance.

### 3.3. Cutting Forces

**Figure 13:**
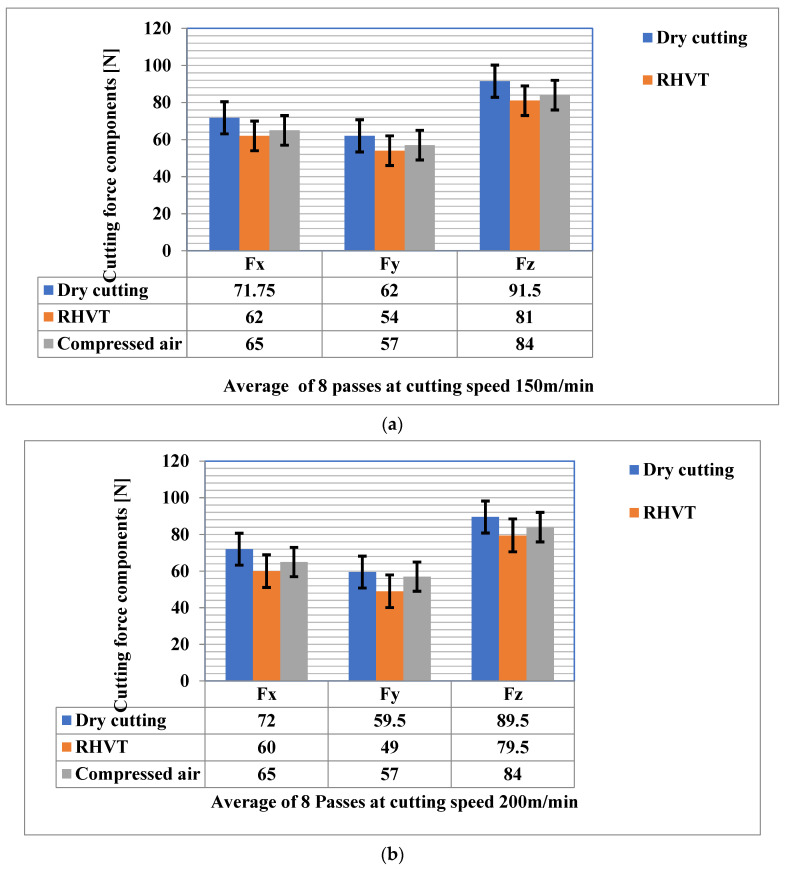
Cutting force components under different cooling strategies at cutting speed of (**a**) 150 m/min, and (**b**) 200 m/min.

Cutting force is one of the most significant parameters, as it leads to a competent machining process by suitable choice of machine, fixtures, cutting tools and operating parameters. Furthermore, to detect breakage and tool wear, tool cutting force monitoring is frequently used. The maximum cutting force components (Fx, Fy and Fz) measured during the tests under dry cutting, compressed air, and RHVT, depending on cutting speeds of 150 m/min and 200 m/min, respectively, with a constant feed rate of 0.12 mm/rev, are shown in Figure 12. RHVT reduces cutting forces better than dry machining owing to the reduction of work material deposition on tool surfaces [36]. This shows that the implementation of RHVT reduces the temperature at various contact interfaces and thus minimizes tool wear, which leads to the reduced workpiece surface roughness. The MF dry cutting strategy generated the highest cutting forces due to the lack of cooling and lubrication, and thus the highest tool wears, as established in Section 3.2. This observation is consistent with the finding of Sikdar and Chen [37] that all cutting forces are augmented in proportion to the increase in tool flank wear. Another important finding in the cutting force analysis was that the MF compressed air strategy resulted in significant tool wear compared to the MF compressed air and RHVT strategies, but even so, cutting force is significantly reduced. This is mainly due to good chip breakability, which thus minimizes tool-chip contact. In general, the cutting forces are reduced as the cutting speed is increased from 150 m/min to 200 m/min (Figure 13). When the cutting speed is increased during machining, the cutting forces are reduced due to the low friction force, owing to thermal softening of the tool rake face at high cutting speeds.

### 3.4. Surface Roughness

**Figure 14:**
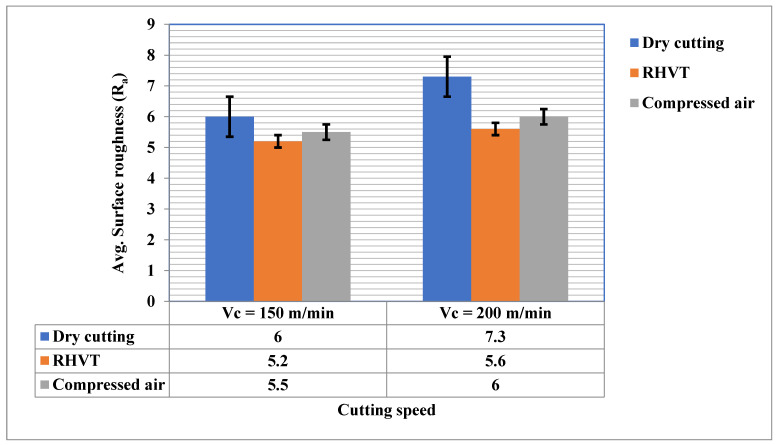
Avg. surface roughness parameter (Ra) for cutting speed 150 m/min and 200 m/min.

The factors affecting surface roughness are BUE, tool wear, cutting conditions, accuracy, chip breaking properties, machine spindle accuracy, etc. These are all components of surface texture. A surface profilometer (Mitutoyo-SJ-400) was employed to measure surface roughness values. To plot the results during an experiment, the average value of three measurements were taken. Regardless of the MT strategy used, the average surface roughness parameter value increases with increasing cutting speed. The main cause of this phenomenon is that tool wear increases as the cutting speed increasesat a cutting speed of 200 m/min compared to 150 m/min, and the surface roughness increases due to the influence of the tool topography [38]. Beneath dry cutting surroundings, the surface roughness values achieved were highest compared to RHVT, which may be due to the absence of any coolant/lubricant used during machining.The value of surface roughness achieved under RHVT environment was low compared to dry conditions [39]. The Ra of the RHVT MF strategy is reduced compared to the dry MF strategy by 17% and 29% for cutting speeds of 150 m/min and 200 m/min, respectively, as illustrated in Figure 14. For all MF strategies tested, the surface roughness values were highest in dry conditions. This is consistent with the results for a diversity of materials, primarily due to the lack of exposure to coolants and lubricants during processing. Compressed air can also reduce surface roughness values due to chip breakability. 

**Figure 15:**
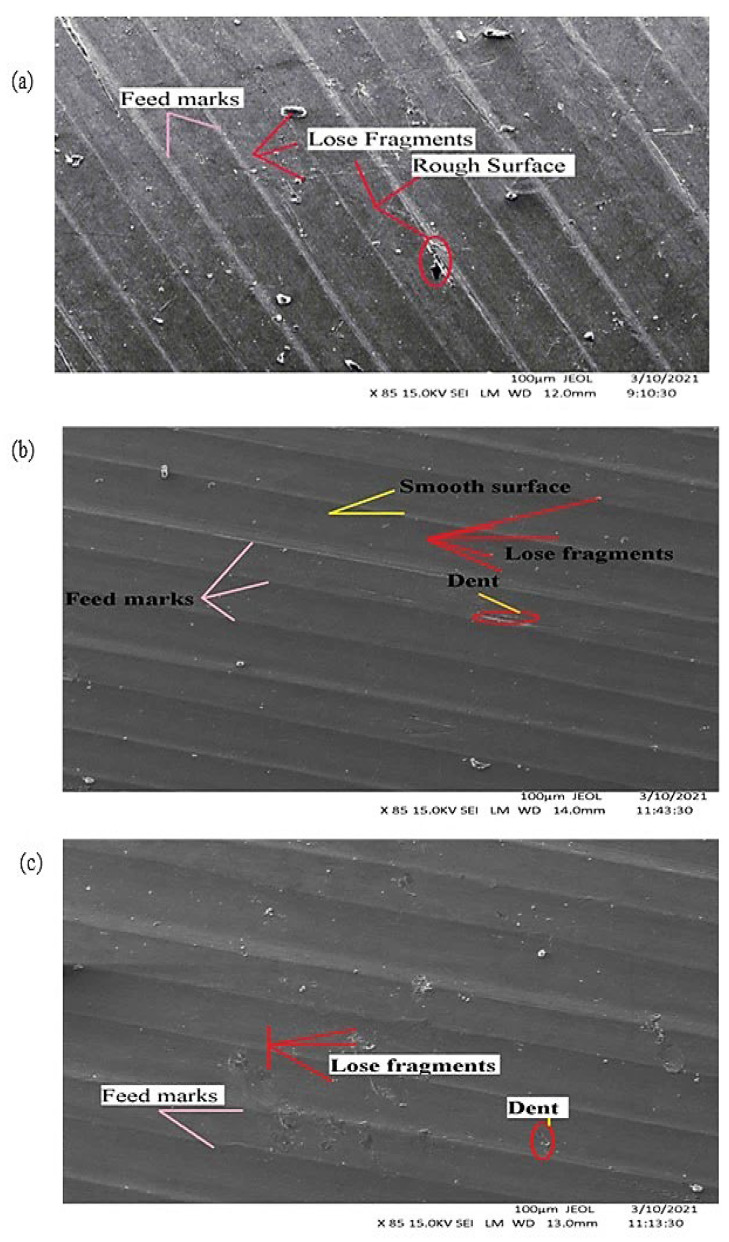
SEM pictures of surface machined at cutting speed of 150 m/min. (**a**) Dry cutting; (**b**) Compressed air; (**c**) RHVT.

The SEM was used to examine the surface profiles of machining surfaces. Figure 15a depicts the SEM of the workpiece surface after machining with the MF dry cutting strategy. Obviously, the surface is quite rough, which is also confirmed by the measured surface roughness (Ra) values for both cutting speeds tested. The roughness of the profile was obtained due to the increase in surface roughness, since during dry cutting the vibration of the machine is dominant in the absence of cooling and lubricating effects. There are noticeable feed marks on the surface, which also confirms that machine vibrations negatively affected the part quality. Additionally, we see a significant number of loose chip fragments on the surface. Figure 15b shows the machined surface under the compressed air method. It did not give satisfactory results and ranked second only to dry machining in terms of surface roughness. All surface irregularities such as dent, feed marks, and loose fragments are visible on the surface. Additionally, the tool wear beneath different cutting methods tested was also found to be in synchronization with workpiece surface finish. Tool wear was observed to be higher for dry cutting followed by compressed air and RHVT. Figure 15c depicts the machined surface beneath the RHVT MF method. Although the surface finish obtained is greater compared to dry cutting, feed marks and loose chip fragments can be seen. The markedly lower quantity of chip fragments and feed marks compared to those from dry cutting suggests the smooth chip flow ensured low surface roughness.

### 3.5. Chip Morphology

**Figure 16:**
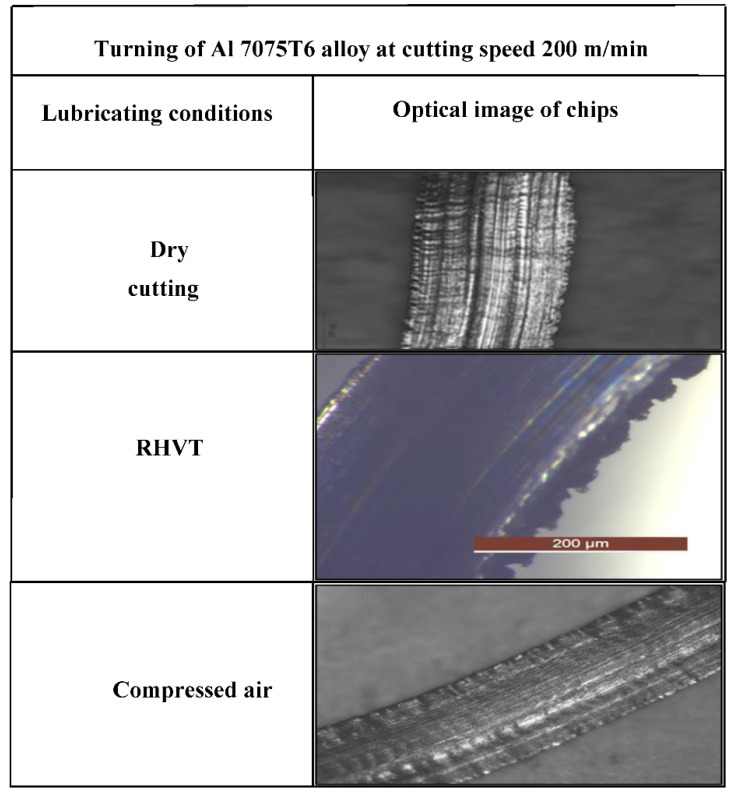
Comparison of chip morphology at different cooling/lubricating machining environments.

The subtraction of excess work material from the workpiece occurs in the form of chips. The chip types depend on nature of the tool, nature of the workpiece, cutting speed, feed rate, dimensions of the tool, friction between the tool and workpiece, and cutting environment factors such as temperature, friction, etc. The study of the morphology of chips is important in machining because it gives an idea of the stability of the cutting process and reveals the mechanical and thermo-chemical influence of the processing materials. The machining of material is highly depended on chips. The machinability of difficult-to-cut materials depends on the chip-forming mechanism, as this affects tool wear and machining surface quality [40]. Particularly, the major problems observed in aluminum alloy machining are chip breakability. In fact, long chips can damage the evacuation system of machines, tools, and work surfaces. Therefore, in order to achieve the consistent machinability of Al 7075T6, which is one of the objectives of this study, chip formation was studied according to various MF strategies. Figure 16 shows chip morphology comparison under dry, compressed air, and RHVT conditions. Fragmented and medium-length chips are generated under the RHVT environment, which indicates chip breakability under this technique. The chips generated at dry are continuous chips. MF dry cutting chips are continuous chips with sharp edges and uneven burrs. Chips produced with the RHVT and MF compressed air strategies were nearly identical, with a mixture of fragmented and medium-length chips, indirectly indicating increased chip breakability.

### 3.6. Microhardness

**Figure 17:**
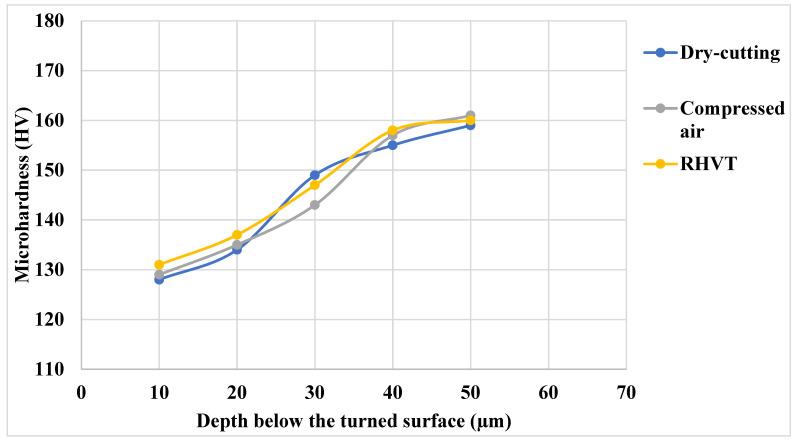
Microhardness (HV) analysis on workpiece under RHVT, compressed air, and dry cutting conditions.

Microhardness is affected by several machining factors. The functionality of a material is affected due to heat generation during machining. Therefore, an effort has been made to evaluate the performance of all three MF strategies on the microhardness of the surface. When measuring microhardness, a Vickers Diamond Indenter (DPH) with penetrometer weighing up to 1000 gm is pressed against the material surface according to ASTME92 standards. The test was performed on a turned surface with a load of 250 gm for 15 s. Microhardness measurement was carried out from the turned surface at a distance (8–10 µm) for the first measurement accompanied by the next measurements after each 10µm. For each cooling condition, 15 microhardness measurements were taken. In Figure 17, any representative value of microhardness at a specific distance is the average of three measurements taken at that point. It is apparent from Figure 17 that, beneath dry conditions, heat does not dissipate well, and therefore it enters the workpiece due to the absence of coolant. This causes extreme damage beneath the turned workpiece. Thus, at 10 µm, a drop in microhardness of approximately 20% can be observed. This was mainly due to the tempering effect of heat in the cutting zone in the case of dry turning. A similar observation was made for RHVT and compressed air, however, the drop in hardness was relatively low compared to dry conditions. This evaluation of microhardness indicated a heat-based attention in the turned workpiece, and thus, the heat-based effect was found to be lowest in RHVT and compressed air compared to dry conditions.

### 3.7. Chip Management

Stringent environmental requirements and regulations force the manufacturing sector to innovate and develop new components using recycled or reused materials. The prerequisites for waste management in the context of sustainable, clean, or environmentally sound production, i.e., zero emissions and no waste, are often considered sensitive research areas [41]. The term “waste” relates to something that is important to us: something around us, and our ability to recycle, reuse, reduce, and eliminate it. Waste management conditions mainly focus on two aspects: the development and subsequent treatment of waste generated during and after operations (reuse, disposal, and disposal of scrap). Likewise, chips produced in turning are directly related to the concept of waste management as they are used to attain closed material flow cycles with minimal or no waste and emissions. Therefore, in this paper, CMM is employed using MF strategies to understand the mechanisms of mechanical material behavior. In this study, factors such as disposed chips, disposal scrap parts, and recycled scrap parts related to zero waste criteria are intentionally pondered. The applicable conditions relating to the implemented model are described below. Data of chips have been used for this purpose during turning of Al 7075T6 alloy.

1. Disposed chips: RDC is computed by Equation (1) where,

- m_c_ is the total mass of chips
- m_cd_ total mass of disposed chips

RDC = m_cd_/m_c_ (1)

2. Recycled scrap part: RRSP is computed by Equation (2) where,

- m_s_ total mass of scrap parts
- m_rsc_ is the total mass of recycled scrap parts

RRSP = m_rsc_/m_s_ (2)

3. Disposal scrap part: RDSP is computed by Equation (3) where,

- m_s_ total mass of scrap parts
- m_ds_ total mass of recycled scrap parts

RDSP = m_ds_/m_s_ (3)

**Figure 18:**
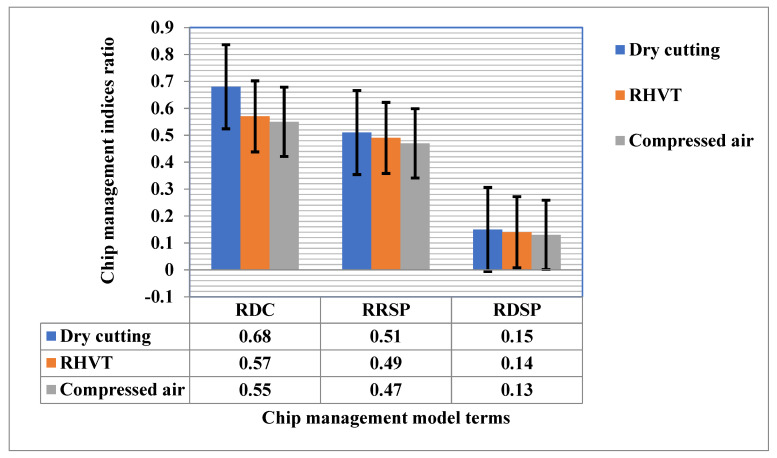
Indices of model based on chip management at different MF conditions.

The dry method obtained high RDC, RRSP, and RDSP, followed by compressed air and RHVT. Dry conditions are insufficient to obtain sustainable chip formation. This is due to the increased thermal conditions at the chip interface. These high-temperature conditions can reduce the quality of chips to be recycled and ultimately contribute to higher chip disposal. The low temperature created by the RHVT and compressed air methods can cause brittleness and chip edge separation. Ease of chip breaking and chip removal from the cutting area contribute to a sustainable and clean production system, as exhibited in Figure 18.

### 3.8. Energy Consumed

Sustainability analysis of turning AA 7075T6 was performed for all MF strategies applied in terms of energy consumption. Energy consumption (*Ec*) was calculated based on Equations (1) and (2) to study the sustainability of the machining process. For pumps, MQLs, and RHVTs, many other components such as compressors and motors also consume energy during rotation. However, this study was limited by the energy consumption needed to remove the material during processing. *Ec* was calculated by taking spindle speeds of 1680 and 1260 rpm at cutting speeds of 200 m/min and 150 m/min when the workpiece diameter is 38 mm.

(4) $$Ec=\frac{Fc\times Vc\times Machining\;Time\;}{60}$$

where, *Ec* = Energy Consumed in kJ and *Fc* = Main Cutting Force in N

(5) Machining Time (min)=60×Machining Length (mm)Spindle Speed in RPM×Feed Rate (mmrev)

Hence, by using the Equation (2), the Equation (1) will become as follows:

$$Ec=\frac{Fc\times Vc\times 60\times Machining\;Length}{60\times Spindle\;Speed\;in\;RPM\times Feed\;Rate}$$

Here, the final *Ec* was calculated in kJ.

Energy consumed was calculated for all three MF techniques, for turning Al 7075T6 alloy as shown in Figure 18. For all analysis of MF techniques, with an increase in cutting speed energy, consumption increases because cutting temperature increases at the elevated cutting speed [42].

**Figure 19:**
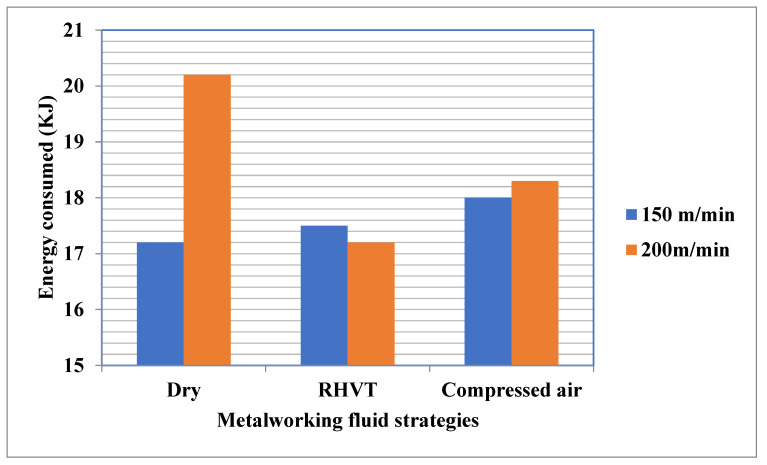
Energy consumed during machining for various MF strategies used at cutting speeds of 150 m/min and 200 m/min.

The maximum energy consumption was observed under dry conditions, followed by compressed air and RHVT. Due to higher friction at the interfaces caused by increased tool wear, the maximum consumption of energy was observed beneath dry conditions. Due to improved cooling in RHVT, a lesser amount of energy consumption was observed. In addition, the absence of any moving mechanical parts in RHVT also facilitates a lower amount of energy consumption during turning processes. As is clearly shown in Figure 19, the energy consumption under the RHVT MF strategy was 4.4% (at a cutting speed of 150 m/min) and 3.2% (at a cutting speed of 200 m/min) compared to dry cutting.

## 4. Conclusions

In this study, to improve the machining performance of Al-5.6Zn-2.5Mg-1.6Cu-0.23Cr-T6 Aerospace alloy, environmentally friendly cooling/lubricant strategies were applied to investigate tool tip temperature, surface roughness, chip morphology, tool wear, microhardness, and surface quality. The following inferences may be drawn from this study.

- The temperature changes obtained through the implementation of the RHVT method have a more positive effect on cutting forces than those obtained viathe dry cutting and compressed air methods. Use of RHVT reduces the cutting temperature between tool and workpiece up to 10% compared to dry turning.
- The RHVT MF strategy reduced Ra compared to the dry MF strategy by 17% and 29% for cutting speeds of 150 m/min and 200 m/min, respectively. For all MF strategies tested; the surface roughness values were highest in dry conditions.
- The VB_max_ decreases with the employment of RHVT more than with in dry and compressed air conditions. The tool flank wear increases with each increment in cutting speed in both cutting conditions. RHVT is effective with each incremental decrease in cutting speed.
- Fragmented and medium-length chips were generated under the RHVT environment, which indicates chip breakability under this technique. The chips generated at dry are continuous chips. MF dry-cutting chips are continuous chips with sharp edges and uneven burrs. Chips produced with the RHVT and MF compressed air strategies were nearly identical, with a mixture of fragmented and medium-length chips, indirectly indicating increased chip breakability.
- The Dry method obtained high RDC, RRSP, and RDSP, followed by compressed air and RHVT.
- Energy consumption under the RHVT MF strategy was 4.4% (at a cutting speed of 150 m/min) and 3.2% (at a cutting speed of 200 m/min) compared to dry cutting.
- Chip morphology analysis proved that the RHVT MF strategy not only facilitates the easy flow of chips on the rake face, but also enhances chip removal from the cutting zone by breaking the chips into segments.

## Figures and Tables

**Table 1:** Mechanical properties of processed material.

Al Alloy	Vicker’s Hardness	Tensile Strength (MPa)	Yield Strength (MPa)	Elongation %
7075-T6	85	567.08	502.14	9.1

**Table 2:** Geometry of cutting insert.

Al Alloy	Rake Angle (°)	Clearance Angle (°)	Lead Angle (°)
7075-T6	3	27	0

## Data Availability

The data presented in this study are available upon request from the corresponding authors.

## Abbreviations

Ranque-Hilsch vortex tube	RHVT
Energy dispersive X-ray spectroscopy	EDS
Scanning electron microscopy	SEM
Chip management model	CMM
Disposal ratio of chips	RDC
Recycled scrap part ratio	RRSP
Disposal scrap part ratio	RDSP
Cutting speed	Vc
Depth of cut	ap
Feed	f
